# Molecular epidemiology of extended-spectrum beta-lactamase-producing *Escherichia coli* in Tunisia and characterization of their virulence factors and plasmid addiction systems

**DOI:** 10.1186/1471-2180-13-147

**Published:** 2013-06-25

**Authors:** Basma Mnif, Hela Harhour, Jihène Jdidi, Faouzia Mahjoubi, Nathalie Genel, Guillaume Arlet, Adnene Hammami

**Affiliations:** 1 Laboratoire de Microbiologie, Hôpital Habib Bourguiba, Sfax, Tunisie; 2 Service de médecine préventive, Hôpital Hédi Chaker, Sfax, Tunisie; 3 Faculté de Médecine, Site Saint-Antoine, Laboratoire de Bactériologie, Université Pierre et Marie Curie-Paris-6, Paris, France; 4 Laboratoire de Microbiologie-Faculté de Médecine de Sfax, Avenue Majida Boulila, 3027, Sfax, Tunisie

**Keywords:** *E. coli*, ESBL, CTX-M-15, Plasmid, Addiction systems, Virulence

## Abstract

**Background:**

Extended-spectrum β-lactamases (ESBLs), particularly CTX-M- type ESBLs, are among the most important resistance determinants spreading worldwide in Enterobacteriaceae. The aim of this study was to characterize a collection of 163 ESBL-producing *Escherichia coli* collected in Tunisia, their ESBL-encoding plasmids and plasmid associated addiction systems.

**Results:**

The collection comprised 163 ESBL producers collected from two university hospitals of Sfax between 1989 and 2009. 118 isolates harbored *bla*_CTX-M_ gene (101 *bla*_CTX-M-15_ gene and 17 *bla*_CTX-M-14_ gene). 49 isolates carried *bla*_SHV-12_ gene, 9 *bla*_SHV-2a_ gene and only 3 *bla*_TEM-26_ gene. 16 isolates produced both CTX-M and SHV-12. The 101 CTX-M-15-producing isolates were significantly associated to phylogroup B2 and exhibiting a high number of virulence factors. 24 (23.7%) of the group B2 isolates belonged to clonal complex ST131. Pulsed-field gel electrophoresis (PFGE) typing revealed a genetic diversity of the isolates. 144 ESBL determinants were transferable mostly by conjugation. The majority of plasmid carrying blaCTX-M-15 genes (72/88) were assigned to various single replicon or multireplicon IncF types and had significantly a higher frequency of addiction systems, notably the VagCD module.

**Conclusion:**

This study demonstrates that the dissemination of CTX-M-15 producing *E. coli* in our setting was due to the spread of various IncF-type plasmids harboring multiple addiction systems, into related clones with high frequency of virulence determinants.

## Background

Extended-spectrum β-lactamases (ESBLs) are among the most important resistance determinants spreading worldwide in Enterobacteriaceae [1,2]. During the 1980s, ESBLs evolved from TEM and SHV broad-spectrum-β-lactamases, frequently associated to *Klebsiella pneumoniae* involved in nosocomial outbreaks. Over the last decade, CTX-M-type ESBLs have increased dramatically, and become the most prevalent ESBLs worldwide, frequently associated to *Escherichia coli*. Among the CTX-M-type ESBLs, CTX-M-15 is now the most widely distributed in *E. coli* which became a major cause of infections in both community and hospitals [1,2]. A few explanations have been proposed on what makes CTX-M-15-producing *E. coli* isolates so successful. First, it has been proposed that the strain virulence background could be involved in this dissemination process. In fact, many reports have shown that CTX-M-15 is closely associated with the international and pandemic uropathogenic O25:H4-ST131 clone, which have specific virulence factors [3,4]. Second, the association of CTX-M-15 with the IncF plasmids, which are well adapted to *E. coli*, may facilitate the spread of this determinant in *E. co*li population [5]. In addition to virulence background and IncF plasmids bearing CTX-M-15, it was recently suggested that the association of various plasmid addiction systems may contribute to the plasmid maintenance in their host [6-8]. An addiction system or a toxin-antitoxin system helps maintain plasmids in bacteria during host replication by killing of plasmid-free cells resulting from segregation or replication defects [9].

In Tunisia, SHV-2 was the first ESBL to be detected, in 1984 from a *K. pneumoniae* clinical isolate [10]. Then, various other types of ESBLs, SHV-12, SHV-2a, CTX-M-15, CTX-M-14, CTX-M-9, CTX-M-16, CTX-M-27 and CTX-M-28 have been reported in different Tunisian hospitals with CTX-M-15 being the most prevalent [11-15]. This study was designed to characterize the ESBL-producing *E. coli* collected in two university hospitals of Sfax, in the southern part of Tunisia and to investigate their virulence background, their ESBL-encoding plasmids and their plasmid addiction systems.

## Methods

### *E. coli* isolates

163 isolates were randomly selected from the collection of ESBL-producing *E. coli* isolates maintained at -80°C in the Microbiology laboratory of Habib Bourguiba hospital. The 163 isolates were collected from the two university hospital of Sfax in Tunisia during the following years: 1989-1990 (6), 2000 (9), 2001 (18), 2002 (9), 2003 (30), 2004 (26), 2006 (36) and 2009 (29). These isolates were obtained mainly from urine (124), but also from blood (20), wound swabs (10), abdominal fluid (5) and sputum (4).

### Antibiotic susceptibility testing

The susceptibility to 16 antimicrobial agents (amoxicillin, amoxicillin + clavulanic acid, ticarcillin, ticarcillin + clavulanic acid, cefalothin, cefoxitin, ceftazidime, cefotaxime, cefepime, gentamicin, amikacin, nalidixic acid, norfloxacin, sulfamethoxzole/trimethoprim and tetracycline) was determined by the disk diffusion method according to the guidelines of the CLSI [16]. All isolates were confirmed for ESBL production using the double disk synergy method.

### Identification of *bla* genes

The resistance genes *bla*_TEM_, *bla*_SHV_ and *bla*_CTX-M_ responsible for the ESBL activity were identified by PCR-sequencing [17]. PCR products were sequenced on ABI Prism 3100 automated sequencer (Applied Biosystems, Foster City, CA) and were analyzed using NCBI BLAST program. (http://www.ncbi.nlm.nih.gov/).

### Strain typing

The phylogenetic group of the ESBL-producing *E. coli* was determined by a multiplex PCR assay [18]. Isolates belonging to phylogenetic group B2 were screened with a previously established PCR-based method to identify the O25b subtype [19]. Furthermore, multilocus sequence typing (MLST) using the scheme of the Institut Pasteur, Paris, France (http://www.pasteur.fr/mlst) was used to confirm that CTX-M-15-producing *E. coli* O25b belonged to the international clone ST131 [19]. Genetic relatedness of the ESBL-producing strains was studied by PFGE following extraction of genomic DNA and digestion with *Xba*I PFGE according to a standard protocol using a GenePath system (Bio-Rad). PFGE banding profiles were compared digitally using Fingerprint II software (Bio-Rad) and relatedness was calculated using the unweighted pair group method with arithmetic mean (UPGMA) algorithm with similarity of bands using the Dice similarity indices. Isolates were considered to belong to the same PFGE cluster if their Dice similarity index was >80% [20].

### Transfer of ESBL resistance determinants and plasmid analysis

Transfer of ESBL encoding genes by conjugation was performed by matting-out assays using *E*. co*li* J53-2 Rif^R^ or *E*. co*li* HB101 Str^R^ as recipient strains. Transconjugants were selected on MH agar containing rifampin (250 μg/mL) or streptomycin (50 μg/mL) plus ceftazidime or cefotaxime (2 μg/ml). When plasmids were not transferable by conjugation, a transformation experiment was assayed. Plasmid DNA obtained using the QIAprep Spin Miniprep kit (Qiagen) were electroporated into *E. coli* DH10B (Invitrogen). Transformants were selected on MH agar plates supplemented with ceftazidime (2 μg/mL) or cefotaxime (2 μg/mL). Plasmids were classified according to their incompatibility group using the PCR replicon-typing scheme described previously [21].

### Detection of virulence factors and plasmid addiction systems

For the ESBL-producing isolates, 17 virulence-associated genes were sought as previously described: *fimH* (type 1 fimbriae), *papG* (P fimbriae adhesion) alleles I, II and III, *papC*, *sfa/focDE* (S and F1C fimbriae), *afa/draBC* (Dr-binding adhesions), *iha* (adhesion siderophore), *hra* (heat(resistant agglutinin), *iutA* (aerobactin receptor), *fyuA* (yersiniabcatin receptor), *cnf-1* (cytotoxic necrotizing factor type 1), *hlyA* (α-hemolysin), *sat* (secreted autoreceptor toxin), *kpsMT* II (group II capsule), *traT* (serum resistance-associated) and *pheR* (phenylalanine tRNA, site of insertion from PAI V) [22].

For *E. coli* recipient strains, seven plasmid addiction system PemK–PemI (plasmid emergency maintenance), CcdA–CcdB (coupled cell division locus) RelB–RelE (relaxed control of stable RNA synthesis), ParD–ParE (DNA replication), VagC-VagD (virulence-associated protein), Hok–Sok (host-killing) and PndA–PndC (promotion of nucleic acid) were sought by PCR as described previously [7].

### Statistical analysis

Comparisons were determined using Pearson’s *x*2 or Fisher’s exact test when appropriate, and a *P* value < 0.05 was considered significant. Statistical analysis was carried out using SPSS version 11.5 for Windows.

## Results

### ESBL characterization and antimicrobial resistance

**Figure 1:**
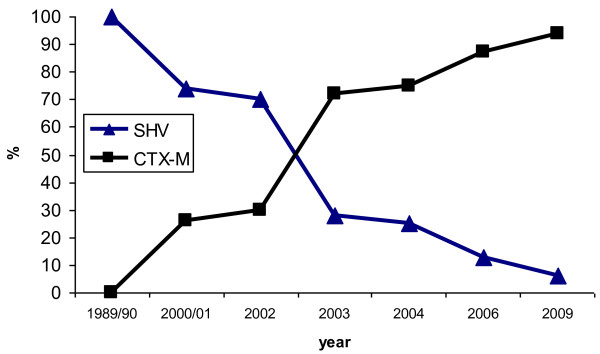
Evolution of SHV and CTX-M ESBL type incidence during the study period.

PCR and sequence analysis revealed that 118 of the 163 (72%) ESBL-positive *E. coli* clinical isolates were CTX-M producers, 101 producing CTX-M-15 and 17 CTX-M-14. 49 isolates produced SHV-12, 9 SHV-2a and only 3, TEM-26. 16 isolates were found to carry both *bla*_SHV-12_ gene and *bla*_CTX-M_ gene (10 *bla*_CTX-M-15_ and 6 *bla*_CTX-M-14_ genes). The occurrence of *bla*_SHV_ genes decreased over time, whereas *bla*_CTX-M_ genes became predominant since 2003 (Figure 1). The ESBL-producing *E. coli* isolates were highly resistant to the aminoglycosides, gentamicin (78%), amikacin (32%), to fluoroquinolones (ciprofloxacin, 62%) and to trimethoprim-sulfamethoxazole (65%).

### Transfer of resistance and plasmid replicon type determination

144 over 179 (80%) ESBL determinants were transferable by conjugation (n = 136) or transformation (n = 8); these encoded CTX-M-15 (n = 88), CTX-M-14 (n = 15), SHV-12 (n = 30), SHV-2a (n = 9) and TEM-26 (n = 2) (Table 1). Only the *bla*_CTX-M_ gene was detected in recipient strains corresponding to *E. coli* isolates harboring both *bla*_SHV-12_ gene and *bla*_CTX-M_ gene, except for one isolate in which the *bla*_SHV-12_ determinant was transferred. 35 ESBL determinants, were non transferable despite repeated conjugation and transformation attempts.

**Table 1:** **Number of replicons according to ESBL type identified in the *E. coli*-recipient strains**

**ESBL type**	**N**	**Replicon type**											
	**All**	F *	**F multireplicon type**					HI2^*^	**I1**	**L/M**	**A/C**	**N**	**ND**
			FII*	**FIA-FIB**	**FII-FIA**	**FII-FIA-FIB**	**FII-FIB**						
All	**144**	**85**	**49**	**5**	**9**	**18**	**4**	**16**	**5**	**14**	**5**	**4**	**15**
**TEM**	**2**	**0**	0	0	0	0	0	**0**	**0**	**2**	**0**	**0**	**0**
TEM-26	2									2			
**SHV**	**39**	**12**	0	3	5	3	1	**14**	**0**	**2**	**5**	**2**	**4**
SHV-2a	9	1					1	2		1	4	1	
SHV-12	30	9		3	5	3		12		1	1	1	4
**CTX-M**	**103**	73*	**49**	**2**	**4**	**15**	**3**	**2**	**5**	**10**	**0**	**2**	**11**
CTX-M-14	15	1	1	0	0	0	0	0	2	3	0	0	9
CTX-M-15	88	72†	48	2	4	15	3	2	3	7†	0	2	2

*ND* not determined.

*: p < 0.05 for CTX-M ESBLs vs. non CTX-M ESBLs.

†: p < 0.05 for CTX-M-15 ESBL vs. other ESBLs.

Fifteen of the 144 ESBL-carrying plasmids (10.4%) were non-typeable for the incompatibility groups sought by the PCR-based replicon typing; 9 of these encoded the CTX-M-14 ESBL, 4 encoded SHV-12 and 2 encoded CTX-M-15. Eighty-five of the 144 ESBL-carrying plasmids (59%) belonged to IncF replicon types. IncF replicons were associated with both SHV and CTX-M ESBL types but were significantly more prevalent in CTX-M-carrying plasmids (CTX-M ESBL type versus SHV, p < 0.001), especially CTX-M-15 ones (Table 1). In fact, 82% of CTX-M-15 genes (72/88) were carried on IncF replicons, 49 on a single FII rep type and, 24 on multiple IncF replicons, occurring in 4 different combinations of IncFII, IncFIA, and/or IncFIB replicons, but FII-FIA-FIB (n = 15) being most frequent (Table 1). HI2 and A/C replicons were associated with SHV ESBL types and L/M and I1 replicons with CTX-M ESBL types (Table 1).

### Strain typing

**Figure 2:**
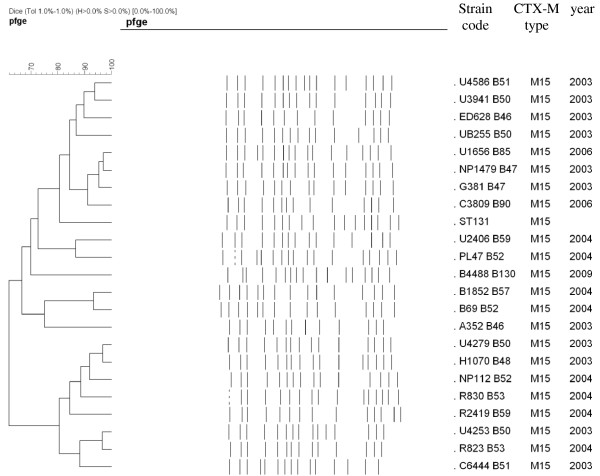
**XbaI-PFGE dendrogram for 22 CXT-M-15-positive *E. coli *isolates from ST131 and a representative ST131 strain from France.**

The 163 ESBL-producing *E. coli* isolates divided among all four major phylogenetic groups: B2 (n = 61), A (n = 54), D (n = 24) and B1 (n = 24). Group B2 was significantly more common among CTX-M-15 producers and group A among SHV producers (Table 2). RfbO25 PCR and MLST revealed that 39% of the group B2 isolates (24/61) and 46.1% of the CTX-M-15-producing B2 isolates (24/52) belonged to the internationally disseminated uropathogenic clone O25:H4-ST131. Of note, these ST131 isolates were recovered mainly in 2003 and 2004 (21 ST131 isolates which accounted for 75% of the B2 isolates) and more rarely in 2006 (2 ST131 isolates) and 2009 (1 ST131 isolate). All of the 163 *E. coli* isolates were subjected to PFGE analysis. However, 15 isolates could not be typed by PFGE. Examination of the 148 PFGE patterns revealed a great genomic diversity with 93 different pulsotypes (62.8%) (Data not shown). 68 isolates corresponded to non-genetic-related isolates, whereas 90 isolates were assigned to 25 minor clonal groups with >80% of similarity; two clusters of 8 isolates, 4 clusters of 4 or 5 isolates and the 19 remaining clusters comprised three or two isolates. The closely related *E. coli* strains were isolated from different wards and years indicating both cross transmission and persistence of some clones in our settings. The SHV-producing isolates were often clonally related, whilst the CTX-M producers were more genetically diverse. Of note, the 22 ST131 strains constituted one large cluster defined at the 61% similarity level; witch was closely tied to a representative strain of the ST131 clonal complex (TN03, [21]). The ST131 cluster, in turn, comprised 6 separate PFGE groups, as defined at the 80% similarity level (Figure 2).

**Table 2:** **Phylogenetic groups of ESBL-producing *E. coli *isolates**

**Phylogenetic group**	**Total**	**CTX-M producers**	**No CTX-M producers**	**CTX-M-15 producers**
Total number	163 (%)	118	45	101
A	54 (33.1)	34	20	26
B1	24 (14.7)	12	12	10
B2	61 (37.4)	55 †	6	52 √
D	24 (14.7)	17	7	13

†: p < 0.0005 for CTX-M B2 producers vs no CTX-M B2 producers.

√: p < 0.0005 for CTX-M-15 B2 producers vs no CTX-M-15 B2 producers.

### Virulence genotyping

The results of the distribution of virulence determinants in *E. coli* isolates in relation with ESBL type and phylogenetic group are reported in Table 3. All the 17 virulence factor genes sought were identified in at least 3 isolates. The most prevalent virulence genes were *fimH* (84.7%), followed by *traT* (73%), *fyuA* (63.8%), *pheR* (60.1%), and *iutA* (50.3%). Isolates belonging to the virulent phylogenetic groups B2 and D had averages of 8.6 and 5.2 virulence factor genes each, respectively, compared with 3 and 3.9, respectively, for isolates belonging to groups A and B1. ESBL-producing *E. coli* isolates belonging to group B2 were significantly more positive for the adhesins *iha*, sfa/foc and papG II and the toxins *sat*, *hylA* and *cnf1* (p < 0.001). Accordingly, distribution rates of most virulence determinants were higher in CTX-M producing *E. coli* isolates than in non-CTX-M producers, as the CTX-M producers especially CTX-M-15 ones were significantly associated to phylogenetic group B2. However, the differences were not significant, except for *papG* allele II, *iha, iutA, sat*, *hylA*, *traT*, and *kpsM II* (Table 3). In fact the B2 isolates with CTX-M-15 had the highest mean score of 8.9 virulence factor genes (Table 3). Of note, the ST131 isolates didn’t exhibit the same virulence profiles. Only five different virulence genes were uniformly present in all 24 ST131 isolates, including *fimH*, *iha*, *sat*, *fyuA*, *iutA* genes and only 16 ST131 isolates belonged to 3 unique virulence profiles. The virulence profiles corresponded inconsistently with PFGE type, suggesting ongoing evolution of virulence genotypes. Moreover, these ST131 isolates were carrying 16 CTX-M-15-plasmids of different types including 7 FIA-FIB-FII, 3 FIA-FII, 3 FII, 2 I1 and one untypeable replicon.

**Table 3:** **Distribution of virulence genes (%) in ESBL-producing *E. coli *isolates**

**Virulence factors**	**Total**	**CTX-M producers**	**Non CTX-M producers**	**CTX-M-15 producers**	**CTX-M-15 B2 producers**	**B2 non-ST131**	**B2 ST131**	**CTX-M-15 B2 ST131producers**
	**N = 163 (%)**	**N = 118**	**N = 45**	**N = 101**	**N = 52**	**N = 37**	**N = 24**	**N = 23**
Total	910	730	180	671	463	332	193	186
Mean	5.58	6.18	4.0	6.64	8.90	8.97	8.04	8.08
Adhesin	3 (1.8)	2 (1.6)	1 (2.2)	2 (1.9)	-	1 (2.7)	-	-
*papG I*								
*papG II*	21 (12.8)	19 (16.1) *	2 (4.4)	19 (18.8)‡	15 (28.8) †	10 (27.2)	5 (20.8)	5 (21.7)
*papG III*	36 (22.0)	30 (25.4)	6 (13.3)	30 (29.7) ‡	25 (48.0) †	24 (64.8) γ	4 (16.6)	4 (17.3)
*papC*	35 (21.4)	29 (24.5)	6 (13.3)	29 (28.7) ‡	25 (48.0) †	25 (67.5) γ	3 (12.5)	3 (13)
*fimH*	138 (84.7)	100 (84.7)	38 (84.4)	85 (84.2)	51 (98.1) †	36 (97.3)	24 (100)	23 (100)
*afa/draBC*	8 (4.9)	4 (3.3)	4 (8.8)	4 (3.9)	2 (3.8)	3 (8.1)	1 (4.1)	1 (4.3)
*sfa/foc*	26 (15.9)	20 (16.9)	6 (13.3)	20 (19.8) ‡	18 (34.6) †	22 (59.4) γ	-	-
*iha*	49 (30.0)	45 (38.1) *	4 (8.8)	43 (42.5) ‡	36 (69.2) †	14 (37.8) γ	24 (100)	23 (100)
*hra*	38 (23.3)	29 (24.5)	9 (20.0)	28 (27.7)	19 (36.5) †	24 (64.8) γ	-	-
Iron uptake	104 (63.8)	78 (66.1)	26 (57.7)	68 (67.3)	49 (94.2) †	33 (89.1)	24 (100)	23 (100)
*fyuA*								
*iutA*	82 (50.3)	65 (55.0) *	17 (37.7)	60 (59.4) ‡	37 (71.2) †	16 (43.2) γ	24 (100)	23 (100)
Toxin	27 (16.5)	24 (20.3) *	3 (6.6)	23 (22.8) ‡	22 (42.3) †	24 (64.9) γ	2 (8.3)	2 (8.6)
*hylA*								
*cnfI*	19 (11.6)	17 (14.4)	2 (4.4)	17 (16.8) ‡	14 (26.9) †	15 (40.5) γ	-	-
*sat*	38 (23.3)	37 (31.3) *	1 (2.2)	35 (34.6) ‡	30 (57.6) †	8 (21.6) γ	24 (100)	23 (100)
Cell protection	119 (73.0)	94 (79.6) *	25 (55.5)	84 (83.2) ‡	40 (76.9)	28 (75.5)	18 (75)	18 (78.2)
*traT*								
*kpsM II*	69 (42.3)	64 (54.2) *	5 (11.1)	59 (58.4) ‡	45 (86.5) †	27 (72.9) γ	23 (95.8)	22 (95.6)
Other *pheR*	98 (60.1)	73 (61.8)	25 (55.5)	65 (64.3)	35 (67.3)	22 (59.4)	17 (70.8)	16 (69.5)

*: p < 0.05 for CTX-M producers vs. non CTX-M producers.

‡: p < 0.05 for CTX-M-15 producers vs. non CTX-M producers.

†: p < 0.05 for CTX-M-15 B2 producers vs. other phylogroup isolates with CTX-M-15.

γ: p < 0.05 for B2 non-ST131 isolates vs. B2 ST131 isolates.

### Addiction systems of ESBL-carrying plasmids

In total, 187 plasmid addiction systems were detected in plasmids encoding ESBLs (mean 1.29, range = 0-4 per recipient strain). *pemKI*, *hok-sok*, *ccdAB* and *vagCD* were the most frequently represented systems (Table 4). None of the plasmids harbored *parDE* or *relBE* and only 5 IncI1 plasmids carried the *pndAC* system. The plasmids bearing CTX-M-15 had more addiction systems than those bearing other ESBLs (mean of 1.62 vs 0.73, respectively, *P* < 0.001). *pemKI*, *vagCD* and *hok-sok* were significantly more prevalent in CTX-M-15-carrying plasmids (Table 4). In addition, the mean number of addiction systems was higher in CTX-M-15-carrying plasmids than in CTX-M-14 carrying ones. Indeed, when the type of replicon was considered, the frequency of addiction systems was the highest in IncF plasmids, which were significantly associated to CTX-M-15-carrying plasmids, and IncI1 ones (mean: 1.90 IncF plasmids and 1.8 IncI1 vs 0.31 other plasmids, *P* < 0.001). IncA/C, IncN, IncHI2 were mostly devoid of addiction systems (Table 2). *pemKI*, *hok-sok*, *ccdAB* and *vagCD* systems were significantly more abundant in IncF plasmids, especially those carrying CTX-M-15 ESBLs (Table 4). When the type of IncF replicons was considered, we remarked that there were no clear relationships between the numbers of the combination of the addiction systems and the different IncF replicon combinations. Nevertheless, the IncFII replicon alone was of the lowest frequency of addiction systems and lacked the *ccdAB* and *vagCD* systems. The FIA-FIB-FII replicon type showed the highest frequency of addiction systems (mean, 2.72), followed by multi-replicon combinations comprising the FIA replicons (Table 4). Statistical analysis showed that *vagCD* is associated with FIA replicons. Moreover, 10 of the 16 (52.5%) CTX-M-15 plasmids carried by ST131 isolates were bearing the *vagCD* systems. In fact, the *vagCD* system was significantly associated to the CTX-M15-producing plasmids carried by ST131 isolates (*P* < 0.0001).

**Table 4:** Nature and number of addiction systems according to ESBL type and replicon type identified in the recipient strains

		**Addiction modules, n**						
**ESBL type**	**n**	***pemKI***^***a***^	***ccdAB***	***hok-sok***^***b***^	***pndAC***	***vagCD***^***c***^	**Total**	**Mean**^**d**^
**All**	**144**	**84**	**29**	**51**	**5**	**18**	**187**	**1.29**
**TEM-26**	**2**	**2**	**0**	**0**	**0**	**0**	**2**	
**SHV**	**39**	12*	**9**	7*	**0**	**3**	**31**	**0.79**
SHV-2a	9	1					1	
SHV-12	30	11	9	7	0	3	30	**1.00**
**CTX-M**	**103**	**70**	**20**	**44**	**5**	**15**	**154**	**1.46**
CTX-M-14	15	6	0	0	2	0	8	0.53
CTX-M-15	88	64	20	44	3	15	143	1.62
**Replicon type**	**n**	*pemKI*^*e*^	*ccdAB*^*f*^	*hok-sok*^*g*^	*pndAC*	*vagCD*^*h*^	Total	Mean^i^
**A/C**	**5**	0	0	0	0	0	0	
**N**	**4**	0	0	0	0	0	0	
**L/M**	**14**	**9**	0	0	0	0	**9**	**0.64**
**IND**	**15**	**4**	0	**1**	0	0	5	**0.33**
**I1**	**5**	**2**	0	0	**5**	**2**	9	**1.80**
**HI2**	**16**	0	0	0	0	**2**	2	**0.12**
**F**	**85**	**69**	**29**	**50**	**0**	**14**	**162**	**1.90**
SHV	12	10	9	6	0	1	26	2.16
CTX-M	73	59	20	44	0	13	136	1.87
**FII**	**49**	**40**	**1**	**32**	**0**	**1**	**74**	1.51
CTX-M-15	48					1		
**FII-FIB**	**4**	**2**	**1**	**2**	**0**	**0**	**5**	1.25
SHV-2a	1	0	0	0	0	0		
CTX-M-15	3	2	1	2	0	0		
**FII-FIA-FIB**	**18**	**15**	**14**	**11**	**0**	**9**	**49**	2.72
SHV-12	3	3	2	3		0		
CTX-M-15	15	12	12	9		9		
**FII-FIA**	**9**	**8**	**8**	**3**	**0**	**4**	**23**	2.55
SHV-12	5	5	4	1		1		
CTX-M-15	4	3	4	2		3		
**FIA-FIB**	**5**	**4**	**5**	**2**	**0**	**0**	**11**	2.20
SHV-12	3	2	3	2				
CTX-M15	2	2	2	0				

^a^*pemKI*: CTX-M vs SHV, p < 0.001; CTX-M-15 vs other ESBLs, p < 0.001.

^b^*hok-sok*: CTX-M vs SHV, p < 0.01; CTX-M-15 vs other ESBLs, p < 0.001.

^c^*vagCD*: CTX-M vs SHV, p =0.23; CTX-M-15 vs other ESBLs, p = 0.03.

^d^ Mean: CTX-M vs SHV, p <0.001; CTX-M-15 vs other ESBLs, p < 0.001.

^e^*pemKI*: IncF vs other plasmids, p < 0.001.

^f^*ccdAB*: IncF vs other plasmids, p < 0.001.

^g^*hok-sok*: IncF vs other plasmids, p < 0.001.

^h^*vagCD*: IncF vs other plasmids, p = 0.08, *vagCD*: IncF and IncI1 vs other plasmids, p = 0.01.

^i^ Mean: IncF vs other plasmids, p < 0.001.

## Discussion

This study provides molecular-epidemiological data on ESBL-carrying *E. coli* isolated in the clinical setting of the two university hospitals of Sfax in Tunisia, in the end of the eighties and the 2000s. This study demonstrates a temporal shift in the prevalence of ESBL types (Figure 1). Thus the CTX-M-type ESBLs have clearly been predominant during the last decade, as has been described worldwide [1,2]. The SHV-2 was the first ESBL to be isolated, in 1984 from a *Klebsiella pneumoniae* isolate in Tunisia [10]. Until the late 1990s, SHV enzymes, especially SHV-12 and SHV-2a, were the most common ESBLs frequently associated with *K. pneumoniae* involved in nosocomial outbreaks in many Tunisian hospitals including our hospital [10,15,23]. In the 2000s, the prevalence of CTX-M increased steadily especially CTX-M-15 type, whereas that of SHV decreased dramatically. In fact, all the 29 studied *E. coli* isolates in 2009 were producing CTX-M-15 ESBL, 2 of these were co-producing SHV-12 ESBL. In accordance with previous reports on distribution of ESBL in Enterobacteriaceae, performed in Tunisia and worldwide, we have shown that the CTX-M-15 ESBL was the most prevalent ESBL in our setting [1,2,12-15]. Recent reports indicate that worldwide dissemination of CTX-M-15 is mediated by clonally related *E. coli* strains, especially a specific clone of phylogroup B2, ST131 [3,4,24]. Accordingly, in the present study, 24/101 (23.7%) of the CTX-M-15-producing strains belonged to clone ST131. *E. coli* ST131 was previously reported in Tunisia in different hospitals since 2005 [13,14,24,25]. One of the Tunisian studies performed in Sousse from May 2005 to May 2006 identified clone ST131 in 23/31 (74%) of CTX-M-15-producing *E. coli* and showed that these 23 isolates had the same pulsotype and the same virulence genotype [14]. However, many reports have demonstrated both homogeneity and considerable diversity in PFGE profiles (<65% similarity) and in virulence gene profiles reflecting the dual phenomenon of recent divergence of the clone from a common ancestor together with ongoing transmission of the clone and ongoing evolution of virulence genotype. Similarly, in the present study the PFGE profiles of the ST131 isolates showed a similarity level of 61% (Figure 2). All theses ST131 isolates expressed the commonly described virulence genes in ST131 clone including *fimH*, *iha*, *sat*, *kpsM*, *fyuA* and *iutA*, however many of these isolates expressed uncommon genes in this clone including *papG* allele II (5 isolates), *papG* allele III (4 isolates), *papC* (3isolates), *afa/draBC* (1 isolate) and *hylA* (2 isolates) (Table 2). Clermont et *al* have shown that the phylogroup B2 pandemic clone ST131 is highly virulent in a mouse model, even though it lacks several genes encoding key virulence factors (Pap, Cnf1, HylA) [26]. Nevertheless, the recent findings of Johnson et *al* point away from ST131 isolates as having higher virulence potential compared with other *E. coli* types in causing invasive infections in a murine sepsis model [27]. Moreover, a recent study have demonstrated that the ST131 clone has a genetic composition that differs from other group B2 strains, and appears to be less virulent than previously suspected [28]. In fact, in the present study, the non-ST131-group B2 isolates, which were significantly associated to CTX-M-15 ESBLs, had a higher frequency of several genes encoding key virulence factors such as adhesins *hra*, sfa/foc, *papC* and papG II and the toxins *hylA* and *cnf1* than had the ST131 isolates (p < 0.01) (Table 3). Surprisingly, unlike most previously published studies, where the ESBL-producing *E. coli* isolates lacked the toxins *hylA* and *cnf1*, in our collection the group B2 isolates especially those carrying CTX-M had a high frequency of *hylA* (42.6%) and *cnfI* (24.5%) (Table 2) [22]. PFGE typing showed polyclonality with sporadic cases and small clusters indicating that the rapid increase of CTX-M-15 producing *E. coli* isolates could be due to the incorporation of *bla*_CTX-M-15_ genes into group B2 clones exhibiting high number of virulence factors as well as ST131. Although ST131 was predominant in 2003-2004, it appeared to be replaced by group B2 strains exhibiting a higher number of virulence factors in 2006 and 2009. The successful spread of CTX-M-15 was reported to be also related to IncF plasmids. The *bla*_CTX-M-15_-carrying plasmid studied here were also assigned to incompatibility groups IncF in 72/88 plasmids and rarely to IncL/M, IncI1, IncN and IncHI2. However, unlike other previous reports, *bla*_CTX-M-14_ was carried often on non-typeable plasmids (9/15) and not on Inc K or IncF replicons [5]. More than half of the IncF plasmids carrying CTX-M-15 belonged to the single FII replicon type (48/72). In fact the IncFII plasmids carrying *bla*_CTX-M-15_ are widely widespread; however, IncF multireplicon plasmids, particularly FII-FIA-FIB, are increasingly reported to be associated to *bla*_CTX-M-15_, which can promote stability in bacteria [2,5]. IncF plasmid types are shown to be well-adapted to proliferate in *E. coli*, but their successful retention in *E. coli* populations may also be attributed to the presence of addictions systems. In deed, here the frequency of addiction system was significantly highest in IncF plasmids particularly multireplicon comprising IncFIA. This is consistent with similar studies conducted in France and recently in UK [7,8]. The *pemKI*, *hok-sok*, and *ccdAB* were previously characterized in IncF replicons; however the *vagCD* system which was reported on *Salmonella* virulence plasmids was surprisingly abundant in IncF CTX-M-15 carrying plasmids in the three studies [9,29]. Of note, the *vagCD* system was significantly associated to CTX-M-15-plasmids carried on ST131 clone in both the present study and the UK one (10/17 (58.8%) and 26/39 (66%); respectively) [8]. In addition, another recent study conducted in South Korea has shown that *vagCD* system was more frequently found in CTX-M-15-producing *E. coli* than in CTX-M-14-producing ones and was surprisingly of high frequency in the main ST11 and ST15 CTX-M-producing-*K. pneumoniae* clones found in South Africa [30]. Moreover, two recent other studies have reported the presence of *vagCD* in IncA/C plasmids carrying two successful carbapenemases NDM-1 and VIM-1 in South Africa and in Canada, respectively [31,32]. Thus this module, VagCD, appears to play a role in spread and maintenance of many successful plasmids and resistant clones worldwide. Finally, plasmid addiction systems present exciting opportunities for the development of novel antibacterial agents targeting pathogens harboring multi-drug resistance plasmids. In fact, the exploitation of addiction systems as an antibacterial strategy via artificial activation of the toxin has been proposed and has considerable potential; however efforts in this area remain in early stages and many challenges are associated with artificial toxin activation [33].

## Conclusion

In conclusion, the present study demonstrates the rapid increase of CTX-M-producing *E. coli* isolates in Sfax-Tunisia and the decline of SHV-type, mediated mainly with the highly conjugative and adapted IncF plasmids carrying *bla*_CTX-M-15_. This study furthermore illustrates that the high prevalence of CTX-M-15 is not only due to the spread of a single clone, mainly the pandemic ST131 clone, but is also associated to the spread of various IncF-type plasmids harboring multiple addiction systems, especially the *vagCD* system, into related clones with high frequency of virulence determinants. The *vagCD* system, which is associated to *Salmonella* virulence plasmids, was significantly associated to the pandemic ST131 clone and has been increasingly reported in various plasmids encoding successful β-lactamases. Based on these findings, larger multicenter studies to determine the contribution of the addiction systems particularly *vagCD* in the maintenance and spread in of many successful multi-drug resistance plasmids worldwide are warranted. Finally the artificial activation of the VagC, the toxin of the VagCD module, could be an exciting opportunity for the development of novel antibacterial agents targeting many clones bearing successful multi-drug resistance plasmids.

## Competing interests

The authors declare that they have no competing interests.

## Authors’ contributions

Conception and design of the study: BM, GA, AH. Laboratory work: BM, HH, NG. Data analysis and interpretation: BM, JJ. Manuscript writing, review, and/or revision: BM, GA, AH. All authors read and approved the final manuscript.
